# Chronic Inflammatory Placental Disorders Associated With Recurrent Adverse Pregnancy Outcome

**DOI:** 10.3389/fimmu.2022.825075

**Published:** 2022-04-22

**Authors:** Emily F. Cornish, Thomas McDonnell, David J. Williams

**Affiliations:** ^1^ Elizabeth Garrett Anderson Institute for Women’s Health, Department of Maternal and Fetal Medicine, University College London, London, United Kingdom; ^2^ Faculty of Engineering Science, Department of Biochemical Engineering, University College London, London, United Kingdom

**Keywords:** chronic placental inflammation, villitis of unknown etiology, chronic histiocytic intervillositis, massive perivillous fibrin deposition, CD8+ T lymphocytes, allograft rejection, fetal growth restriction, stillbirth

## Abstract

Chronic inflammatory placental disorders are a group of rare but devastating gestational syndromes associated with adverse pregnancy outcome. This review focuses on three related conditions: villitis of unknown etiology (VUE), chronic histiocytic intervillositis (CHI) and massive perivillous fibrin deposition (MPFD). The hallmark of these disorders is infiltration of the placental architecture by maternal immune cells and disruption of the intervillous space, where gas exchange between the mother and fetus occurs. Currently, they can only be detected through histopathological examination of the placenta after a pregnancy has ended. All three are associated with a significant risk of recurrence in subsequent pregnancies. Villitis of unknown etiology is characterised by a destructive infiltrate of maternal CD8+ T lymphocytes invading into the chorionic villi, combined with activation of fetal villous macrophages. The diagnosis can only be made when an infectious aetiology has been excluded. VUE becomes more common as pregnancy progresses and is frequently seen with normal pregnancy outcome. However, severe early-onset villitis is usually associated with fetal growth restriction and recurrent pregnancy loss. Chronic histiocytic intervillositis is characterised by excessive accumulation of maternal CD68+ histiocytes in the intervillous space. It is associated with a wide spectrum of adverse pregnancy outcomes including high rates of first-trimester miscarriage, severe fetal growth restriction and late intrauterine fetal death. Intervillous histiocytes can also accumulate due to infection, including SARS-CoV-2, although this infection-induced intervillositis does not appear to recur. As with VUE, the diagnosis of CHI requires exclusion of an infectious cause. Women with recurrent CHI and their families are predisposed to autoimmune diseases, suggesting CHI may have an alloimmune pathology. This observation has driven attempts to prevent CHI with a wide range of maternal immunosuppression. Massive perivillous fibrin deposition is diagnosed when >25% of the intervillous space is occupied by fibrin, and is associated with fetal growth restriction and late intrauterine fetal death. Although not an inflammatory disorder per se, MPFD is frequently seen in association with both VUE and CHI. This review summarises current understanding of the prevalence, diagnostic features, clinical consequences, immune pathology and potential prophylaxis against recurrence in these three chronic inflammatory placental syndromes.

## Introduction

Miscarriage, preterm birth, fetal growth restriction and stillbirth are complex multifactorial disorders that carry immense medical, psychological and economic impact (1–3). There are national and global imperatives to reduce their frequency (4, 5). Although almost half of intrauterine fetal deaths remain unexplained (6), a significant proportion (approximately one-third) are attributed to placental disorders detectable at routine histopathological examination (7). Within this category are the three conditions that form the subject of this review: villitis of unknown etiology (VUE), chronic histiocytic intervillositis (CHI) and massive perivillous fibrin deposition (MPFD). They are distinguished from other chronic inflammatory lesions of the placenta, such as chronic deciduitis and chronic chorioamnionitis, by their striking associations with adverse obstetric outcomes and recurrent pregnancy loss (8). It is their recurrent nature that makes them particularly important for patients and clinicians, despite their relative rarity. VUE affects 5-15% of placentas (9), CHI 0.2% (10), and MPFD 0.5% (11). The underlying disease mechanisms are poorly understood and there are no biomarkers or treatment protocols that can reliably predict or prevent recurrence in a subsequent pregnancy (12, 13).

**Table 1** summarises the definitions, diagnostic criteria, grading systems and pregnancy outcomes for each of these disorders. It should be emphasised that these are not universally applied; some studies discussed in this review use different or unspecified definitions, and there is no universal consensus on diagnostic criteria.

**Table 1:** Definitions, diagnostic criteria, severity grading and key obstetric outcomes for three chronic inflammatory placental disorders associated with recurrence risk: villitis of unknown etiology (VUE), chronic histiocytic intervillositis (CHI) and massive perivillous fibrin deposition (MPFD).

	Villitis of unknown etiology (VUE)	Chronic histiocytic intervillositis (CHI)	Massive perivillous fibrin deposition (MPFD)
Definition	Infiltration of maternal CD8+ T lymphocytes into the chorionic villi, with proliferation of activated fetal macrophages (Hofbauer cells) (9, 14, 15)	Presence of a CD68+ maternal histiocytic infiltrate in the intervillous space, accompanied by varying degrees of fibrin deposition (16–18)	>25% of the intervillous space occupied by fibrin, leading to villous engulfment, atrophy and sclerosis (12, 19–21)
Diagnostic criteria	1. Villous infiltrate as above; 2. Absence of an infectious cause (9)	1. 80% of intervillous immune cells are CD68+ histiocytes; 2. ≥5% of the intervillous space occupied by the infiltrate; 3. Absence of clinical or histopathological signs of infection (22)	As above
Grading of lesion extent/ severity	Low-grade: involvement of <10 villi per focus High-grade: involvement of ≥10 villi per focus (9)	Low-grade: 5-50% of the intervillous space occupied by histiocytes and/or histiocyte-associated fibrin High-grade: ≥50% of the intervillous space occupied by histiocytes and/or histiocyte-associated fibrin (10, 23, 24)	Classic (severe): ≥3mm fibrin encasing the basal villi of the entire maternal surface Transmural (moderate): extending from the maternal surface towards the fetal surface and surrounding ≥50% of villi on ≥1 slide Borderline (mild): as with transmural, but only 25-50% villi affected (19)
Pregnancy outcome	Live birth in >90%; has also been associated with recurrent pregnancy loss in small series (25–27) Fetal growth restriction in 23-66% (25, 28–30)	Live birth: 55% Stillbirth: 38% Fetal growth restriction: 72% (22)	Live birth: 83-85% Stillbirth: 15-17% (11) Fetal growth restriction: wide range reported; precise risk uncertain (11, 31, 32)
Recurrence	15-55% (27, 33, 34)	25-100% (22, 35–37)	10-80% (11, 19, 21, 31, 38, 39)

**Figure 1:**
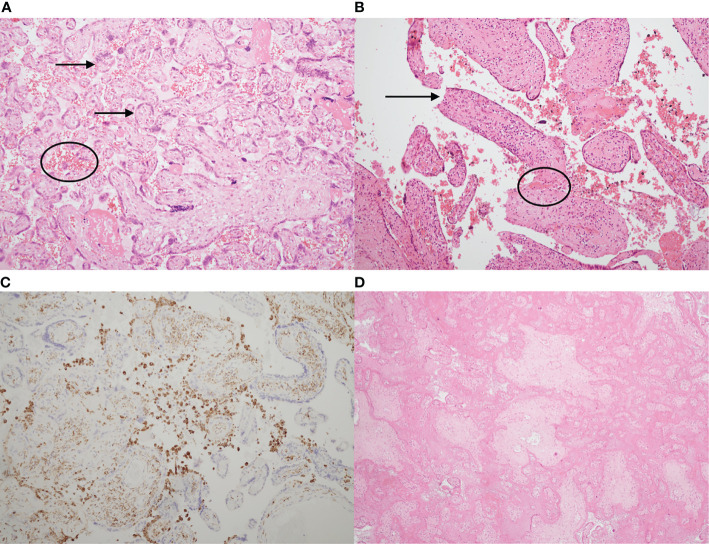
Typical histological appearances of villitis of unknown etiology (VUE), chronic histiocytic intervillositis (CHI) and massive perivillous fibrin deposition (MPFD). **(A)** Haematoxylin & eosin stain of a slide showing normal placenta at 35 weeks’ gestation, with normal chorionic villi (arrows) and maternal erythrocytes in the intervillous space (circled). **(B)** Haematoxylin & eosin stain demonstrating VUE. There is an excess of stromal T lymphocytes, villous agglutination (circled) and loss of trophoblast integrity (arrow). **(C)** Chronic histiocytic intervillositis. Immunohistochemistry demonstrates presence of CD68+ maternal histiocytes (brown) in the intervillous space. **(D)** Massive perivillous fibrin deposition (MPFD), with >95% of the intervillous space obliterated by fibrin. There is also villous agglutination and loss of nuclear integrity. **(A,B)**, x10. **(C)**, x2. **(D)**, x4.

**Figure 1** shows the characteristic histological appearances of VUE, CHI and MPFD.

## Placental Inflammation In Healthy And Pathological Pregnancy

### Maternal Tolerance of the Semi-Allogeneic Fetus

Healthy term pregnancy requires maternal immunological tolerance of the semi-allogeneic fetus. This state is established rapidly in the decidua, where maternal innate immune cells [including macrophages, dendritic cells, natural killer cells and T cells (40–42)] are in direct contact with fetal extravillous trophoblast (EVT). The invading EVT infiltrates through the decidua into the myometrium, driving maternal recognition of fetal antigens. Successful implantation depends on an inflammatory milieu, which is established through production of pro-inflammatory cytokines by the decidual immune system (43, 44). Once implantation has occurred, the balance shifts. Immune crosstalk mediated by human leukocyte antigen (HLA)-G, which is expressed on the EVT (45), induces expansion of Foxp3+ regulatory T cells (T-regs) and tolerogenic macrophage and dendritic cell subsets. Anti-inflammatory cytokines such as IL-10 predominate and cytotoxic effector T cells are suppressed (46). This tolerogenic environment is maintained throughout the second and third trimesters under the influence of hormones, cytokines and soluble mediators (44).

At term gestation, an abrupt return to the pro-inflammatory state triggers the onset of labour. Circulating maternal neutrophils and macrophages are recruited to the decidua through chemotaxis and infiltrate the chorio-amniotic membranes, stimulating myometrial contraction and membrane rupture (47–49). Aberrant activation of this pathway in early gestation can lead to spontaneous preterm labour (49).

### Placental Histopathology in Normal Pregnancy

The defining histological features of VUE, CHI and MPFD can all be found, to varying extents, in healthy pregnancy with normal fetal growth. Chronic villitis has been reported in up to 33% of term placentas, yet the vast majority have a normal clinical outcome (25, 50). Its prevalence increases with advancing gestational age. This suggests that as tolerance declines near term, there may be a loss of the regulatory mechanisms that protect the villi from infiltration by maternal T-effector cells (9). Similarly, the presence of maternal CD68+ histiocytes occupying up to 5% of the intervillous space can be associated with normal outcome (22, 51). Foci of fibrin deposition are found in all placentas, increasing with advancing gestational age, and perivillous fibrin deposition is a physiological response to trophoblast injury (19, 20, 52). Whether VUE, CHI and MPFD represent exaggerated versions of physiological responses, or distinct disease entities, remains unclear.

Determining the true prevalence of these features in normal pregnancies is difficult. It requires a prospective approach, access to specialist perinatal pathology services, and precise, objective case definitions. Retrospective studies of placentas sent to histopathology for clinical indications are influenced by selection bias and will invariably overestimate their frequency (53).

Two studies have attempted to overcome this challenge. In a prospective, unselected analysis of 1,153 women delivering at 34-43 weeks’ gestation, VUE, CHI and MPFD were detected in 35/935 (3.7%), 2/935 (0.2%) and 2/935 (0.2%), respectively, of pregnancies with normal obstetric and perinatal outcomes (53). The strengths of this study include its prospective design and the fact that the reporting pathologists were completely blinded to clinical outcomes. However, the small numbers of cases of chronic inflammatory conditions detected, the lack of any histological grading and the inclusion of preterm deliveries limit its generalisability (53).

In another study of 946 placentas from term deliveries of non-growth-restricted infants (birthweight 10-90^th^ centile), 283/946 (30%) had a chronic inflammatory lesion and 176/946 (19%) had VUE. However, the majority of these were mild, whereas only 11/927 (1.2%) had high-grade VUE (54). The disparities in rates of VUE between the two studies probably reflect (1): The fact that the second study, which reports significantly higher prevalence, includes only term deliveries; and (2) Inconsistent definition and severity classification of VUE – the first study does not state its case definition for VUE. It is possible that only high-grade lesions were reported, which would make its prevalence (35/935, 3.7%) comparable to that reported in the second study (11/927, 1.2%) (53, 54).

These studies prove that chronic placental inflammation can occur in healthy pregnancy. They also highlight the uncertainties generated by lack of universal diagnostic criteria and severity grading systems. The key question is how to define the transition from physiology to pathology, and the mechanism by which this is provoked.

### Chronic Placental Inflammation and Autoimmune Disease

VUE, CHI and MPFD have been reported in conjunction with several immune-mediated diseases, including systemic lupus erythematosus (SLE), Sjögren’s syndrome and autoimmune thyroid disease (12, 35, 55, 56). In particular, they are associated with 2 other disorders with significant implications for pregnancy outcome: obstetric antiphospholipid syndrome (APS) and fetal-neonatal alloimmune thrombocytopenia (F-NAIT). Salafia et al. studied a cohort of 13 women with recurrent pregnancy loss due to APS and found VUE in 4/13 (31%) and MPFD in 6/13 (46%) of their placentas (57). In another retrospective series of 38 CHI pregnancies in 12 women, 4/12 (33%) affected women had APS (58). The over-representation of VUE and CHI in F-NAIT, a rare disorder affecting 1 in 1,000 live births (59), is especially pronounced. In one multi-centre case-control study, VUE and CHI were detected in 10/27 (37%) and 11/27 (41%) of F-NAIT placentas, respectively (60).

Antiphospholipid syndrome, in which maternal autoantibodies are associated with thrombosis and recurrent pregnancy loss, accounts for up to 25% of recurrent miscarriage (61). It is also strongly associated with fetal growth restriction (FGR), pre-eclampsia and stillbirth (62). APS is diagnosed according to international consensus criteria, summarised in **Table 2** (63).

**Table 2:** Updated international consensus classification criteria for the diagnosis of antiphospholipid syndrome (APS). APS is diagnosed when at least 1 clinical criterion and at least 1 laboratory criterion are met (63).

Clinical criteria	Vascular thrombosis	One or more episode(s) of arterial, venous or small vessel thrombosis
	Pregnancy morbidity	a) One or more unexplained death(s) of a morphologically normal fetus at ≥10 weeks’ gestation; or b) One or more preterm birth(s) of a morphologically normal baby <34 weeks’ gestation due to severe pre-eclampsia, eclampsia or placental insufficiency; or c) Three or more consecutive spontaneous miscarriages before 10 weeks’ gestation (in the absence of an anatomical, hormonal or chromosomal cause)
Laboratory criteria	Any of the following present on two or more occasions, 12 weeks apart: 1. Lupus anticoagulant 2. Anti-cardiolipin antibody 3. Anti-beta-2-glycoprotein-1 antibody	

Despite the clear association between APS and adverse pregnancy outcome, these criteria encompass a wide range of clinical phenotypes and the mechanism by which pregnancy loss occurs remains controversial. Early and late pregnancy loss have different aetiologies, reflecting the major adaptations in decidual immunity and utero-placental circulation that occur as pregnancy progresses. Two key mechanisms identified in APS are:

1. Autoantibody-driven thrombosis of the utero-placental circulation: levels of annexin-V, a potent anticoagulant, are significantly reduced in placental villous tissue from women with APS. Exposure of cultured trophoblast and endothelial cells to antiphospholipid antibodies leads to decreased annexin-V expression and accelerated thrombosis (64).
2. Complement-mediated pregnancy loss: in mice injected with human antiphospholipid antibodies, administration of a C3 convertase inhibitor (which blocks classical complement activation) protects offspring from FGR and intrauterine death (65). In women with antiphospholipid antibodies, complement activation in the first trimester is predictive of adverse pregnancy outcome (including FGR and stillbirth) (66). Deposition of the complement degradation product C4d is a hallmark of antibody-mediated rejection in solid organ transplants (67–69). It is generated through activation of the classical complement pathway in response to specific antibody-antigen binding. C4d deposition is significantly more common in placentas from women with antiphospholipid antibodies than those without (70).

APS provides an attractive paradigm for investigating an immunological aetiology in VUE, CHI and MPFD, given that all four disorders cause recurrent pregnancy loss. C4d deposition is also seen in VUE (71), CHI (72) and MPFD (73). However, there are several caveats to this comparison. Firstly, the pregnancy losses associated with VUE, CHI and MPFD usually occur in the second and third trimester, whereas APS primarily causes early first-trimester miscarriage (74). Secondly, although they overlap, the majority of women with VUE, CHI and MPFD do not have APS (56). Thirdly, C4d deposition is not specific to these conditions and has also been identified in unexplained recurrent miscarriage (75). Finally, there are clearly additional mechanistic factors involved given that neither anti-thrombotic therapy (low-molecular weight heparin) (76) nor non-specific immune suppression (hydroxychloroquine) can completely alleviate the risk of obstetric complications in APS (77).

Discordance of VUE, CHI and MPFD has been reported in dizygotic twin pregnancies (78–80). This suggests that fetal genetics contribute to the pathogenesis, with a specific feto-placental antigen stimulating maternal immune “rejection” of the placenta, rather than a general failure of tolerance at the decidua. However, other evidence implies that the primary problem is maternal, particularly in the case of CHI. Firstly, CHI carries a recurrence rate of up to 100% and worsens in successive pregnancies (36, 81–83). Secondly, when women with recurrent pregnancy loss due to CHI undergo IVF with their own oocyte and their partner’s sperm, followed by implantation into a surrogate, CHI does not recur (84). The true underlying mechanism may therefore incorporate a double hit of both fetal immunogenetics and inappropriate maternal immune reactivity: perhaps a feto-placental antigen expressed in the first pregnancy “primes” a mother with a pre-existing propensity towards autoimmune disease to reject all subsequent pregnancies.

### Chronic Placental Inflammation and Infection

Placental villitis is evident in response to various infections (85). In the absence of proven infection, VUE should be considered (86). While unidentified infections could be responsible for what we currently term VUE, this seems unlikely given that infectious villitis and VUE differ in both histological and clinical parameters. **Table 3** summarises the key differences between infectious villitis and VUE.

**Table 3:** Comparison of clinical and histological features in infectious villitis and villitis of unknown etiology (VUE) (9).

	Infectious villitis	VUE
Prevalence	0.1-0.4%	5-15%
Histological features	Diffuse distribution Histiocytic villitis Presence of villous plasma cells Haemosiderin deposition Viral inclusion bodies	Focal or patchy distribution Lymphohistiocytic villitis Absence of plasma cells Absence of haemosiderin Absence of viral inclusion bodies
Symptomatic maternal infection during pregnancy	Common	Rare
Association with maternal autoimmune disease	Rare	Common
Recurrence	Rare	Common

Similarly, the term “CHI” is used interchangeably with “chronic intervillositis of unknown etiology” (CIUE), defined by Bos et al. as an infiltrate of CD68+ mononuclear cells occupying ≥5% of the intervillous space “in the absence of clinical or histopathological signs of infection” (22). This is what distinguishes CHI from the many infectious causes of intervillositis – for example, Plasmodium falciparum (87), cytomegalovirus (88), dengue virus (89), Zika virus (90), parvovirus B19 (16), SARS-CoV-2 (91), Chlamydia psittaci (92) and Listeria monocytogenes (12, 55, 93, 94).

The association of all three conditions with SARS-CoV-2 has provided the opportunity to study rates of VUE, CHI and MPFD in thousands of infected placentas. This is in stark contrast to the handful of case reports describing their occurrence in certain infections prior to the COVID-19 pandemic. A systematic review of placental pathology in 1,008 pregnant women with SARS-CoV-2 infection identified chronic inflammatory pathology (including chronic villitis) in 26% and increased perivillous fibrin in 33% (95). Pregnancy outcomes were not reported in this study, meaning that the clinical consequences of this chronic inflammation are unclear (95). However, large cohort studies and meta-analyses have shown that SARS-CoV-2 infection in pregnancy is associated with significantly higher rates of preterm birth, stillbirth, perinatal and neonatal morbidity and mortality (96–99).

**Figure 2:**
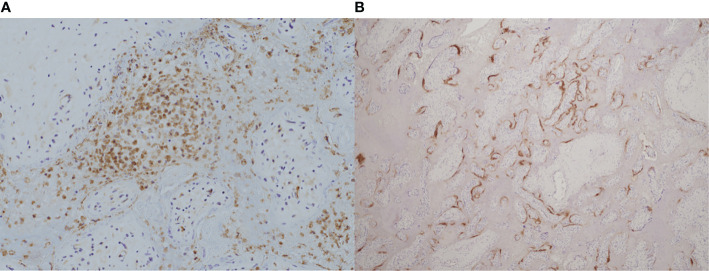
Coexistence of chronic histiocytic intervillositis (CHI) with SARS-CoV-2 infection in a placenta from a late miscarriage at 20 weeks’ gestation. **(A)** Immunohistochemical staining demonstrates CD68+ histiocytes (brown) in the intervillous space. **(B)** Positive expression of SARS-CoV-2 nucleocapsid protein (brown) by immunohistochemistry within the syncytiotrophoblast membrane of the same placenta. **(A)**, x20. **(B)**, x4.

More specifically, a combination of CD68+ histiocytic intervillositis with chronic villitis, perivillous fibrin deposition and trophoblast necrosis has been described in case reports and small series of placental SARS-CoV-2 (91, 100–102). One group studied 39 placentas from SARS-CoV-2-positive mothers and found that all four that stained positive for the virus also had CHI, MPFD, chronic villitis and an unusual CD20+ B cell component in the intervillous infiltrate. This pattern was seen irrespective of severity of maternal symptoms and correlated with fetal distress (103). **Figure 2** demonstrates coexistent CHI and SARS-CoV-2 infection.

High SARS-CoV-2 viral loads in the placenta are unsurprising given that it enters host cells using the angiotensin-converting enzyme 2 (ACE-2), which is highly expressed in the decidua and trophoblast (104). But why would SARS-CoV-2 trigger intervillositis? Infection with other single-stranded RNA viruses such as dengue virus and SARS-CoV-1 is also associated with chronic intervillositis, but the mechanism by which this occurs is unknown (89, 105). It is uncertain whether the accumulation of maternal histiocytes in the intervillous space, adjacent to the SARS-CoV-2 nucleocapsid-positive syncytiotrophoblast, represents a specific response to that particular virus or a non-specific component of the maternal immune response to intra-placental infection.

The detection of this new viral aetiology for villitis and intervillositis highlights the critical importance of accurately excluding an infectious cause when making the diagnosis of VUE or CHI. This remains a conflicted area. One study showed no evidence of increased receptor specificity for common viral epitopes in T cells isolated from VUE placentas, concluding that VUE is more likely to represent a primary allograft rejection response (106). However, another group used viral nucleic acid enrichment combined with metagenomic sequencing to investigate occult viral infection in 20 placentas diagnosed with VUE, and detected at least 1 virus in 65% of them, compared to 35% of control placentas without chronic inflammatory lesions (107). Whatever the pathophysiology of infection-associated villitis or intervillositis, thorough placental examination and/or maternal serology should be used to rule out infection, as the infection-associated cases do not appear to recur.

In the absence of an infectious cause, attention should focus on an immune-mediated pathophysiology. Parallels with transplant immunology have led some groups to describe VUE, CHI and MPFD as analogous to maternal “rejection” of the fetal semi-allograft (13, 72, 83, 108, 109). However, unlike solid organ allografts, serial sampling of the placenta during pregnancy is impossible given the associated risks to both mother and fetus. The diagnosis of VUE, CHI or MPFD therefore rests entirely on placental histology from a single, postnatal timepoint. This makes it particularly difficult to elucidate the sequence of events that culminates in villitis, histiocytic infiltration of the intervillous space or deposition of perivillous fibrin.

### Insights From Animal Studies

The inherent challenges of studying the human placenta in “real time” have prompted the use of alternative strategies, such as primary trophoblast cultures and commercially available cell lines (110–112). However, these have their own limitations: primary cytotrophoblast cells typically fail to replicate long-term in culture and cell line expression profiles diverge significantly from the trophoblast tissues they are intended to mimic (110, 112, 113).

As a result, animal studies have proved essential in building our understanding of human placental function and disease. There is enormous inter-species structural diversity in the placenta. Rodent, rabbit, primate and human placentas are classified as haemochorial, in which maternal blood is in direct contact with fetal-origin trophoblast (114). Of this category, mice have obvious practical advantages for laboratory work (they are small, with short gestation periods and large litter sizes) and have become the most widely used. Murine models of alloimmune disorders have provided valuable insights into pathogenesis and treatment, demonstrating that the neonatal Fc receptor (FcRn) is necessary for transport of maternal IgG into the fetal compartment in F-NAIT (115) and that activation of the classical complement pathway correlates with fetal death in APS (116).

Although they share haemochorial placentation, other critical discrepancies between murine and human placental development and immunology compromise the translational value of mouse models (117). The most important difference is in their capacity for trophoblast invasion. Successful human placentation depends on deep infiltration of the outer myometrium by migrating extravillous trophoblasts, which remodel maternal spiral arteries into dilated, low-pressure vessels to maximise placental blood flow. Inadequate invasion and remodelling leads to fetal growth restriction, pre-eclampsia and fetal death (118). Decidual natural killer (dNK) cells, which are the most abundant maternal leukocyte population at the time of implantation, play an essential role in orchestrating this invasion, through expression of killer cell immunoglobulin-like receptors (KIR) that interact directly with non-classical HLA on invading EVT (119–121). However, murine NK cells do not express KIR and trophoblast invasion is far more superficial, limited to the decidua basalis (117).

In female mice, pre-conception exposure to paternal antigen in seminal fluid stimulates a specific effector CD8+ T-cell response (122). However, this response is transient: if the mouse subsequently becomes pregnant with an embryo that expresses the same antigen, these specific T-cell populations undergo clonal deletion to maintain tolerance to the developing fetus (123). By contrast, in humans, fetal-specific T cells are detectable several years after delivery (83, 124, 125). This persistence of anti-fetal T cells could account for the recurrence and worsening clinical severity observed in CHI and VUE, but murine models could not support investigation of this hypothesis.

Non-human primates share greater parallels with humans in terms of the structure and immunology of the maternal-fetal interface (126). The rhesus macaque has been particularly well studied (127–129). Although they do not exhibit trophoblast invasion to the same degree as humans (126), rhesus macaques express non-classical HLA-G homologs (130), possess a large population of dNK cells (131) and can develop pre-eclampsia (132, 133). Their clinical and histological response to certain viral infections in pregnancy is closely correlated with that seen in humans (134). Rhesus macaques infected with Zika virus during pregnancy show high rates of fetal loss with histiocytic intervillositis and massive perivillous fibrin deposition on placental histology (90, 135–137). Although idiopathic chronic placental inflammation has never been documented in primates, studying infection-associated CHI/MPFD in these models could reveal new insights into the underlying mechanisms involved.

Despite their potential, the ethical, logistic and financial restrictions on availability and maintenance of female rhesus macaques have limited translation of these shared features into reliable models of human placental disease (114).

The recent development of epithelial trophoblast organoids, which recapitulate the 3-dimensional villous architecture, HLA expression profiles and endocrine functions of the human placenta, could transform the study of placental development and disease (138). As with the other models described, the current organoids have intrinsic limitations in terms of their translatability to *in vivo* placental function in general and to chronic inflammatory placental conditions in particular. They are derived from first-trimester placental tissue [whereas the majority of VUE- and CHI-related pregnancy loss occurs in the third trimester (9, 22)], lack vascularisation, and develop without the influence of the decidual and fetal immune systems (139). Development of third-trimester placental organoids has so far proved impossible (140), but would represent a vital step towards the elusive *in vitro* model of the term human placenta in health and disease.

The following sections will summarise evidence for an immunological aetiology in VUE, CHI and MPFD and highlight current challenges in the efforts to understand how these conditions cause recurrent adverse pregnancy outcome.

## Villitis Of Unknown Etiology (VUE)

### VUE: Definition, Prevalence, and Diagnosis

As mentioned above, VUE is evident in 5-15% of third-trimester placentas, with increasing frequency towards term (9, 141, 142). A maternal lymphohistiocytic infiltrate consisting primarily of CD8+ cytotoxic T cells invades the villous stroma, causing destructive inflammation that can lead to villous sclerosis and necrosis. In severe cases the villous inflammation can progress to “obliterative fetal vasculopathy”, in which complete luminal occlusion of fetal stem villi leads to avascular villi and fetal hypoxia (143). There is also expansion of activated fetal macrophages (Hofbauer cells) within the villi (9, 12, 14, 15). VUE is classified according to the spatial pattern of villous involvement: distal, proximal or basal villi. It can be high-grade (>10 contiguous villi affected in more than one section) or low-grade (≤10 villi affected in any single focus) (9, 144). Key factors that distinguish it from infectious villitis include its non-uniform patchy distribution within the placental parenchyma and the absence of neutrophils and viral inclusions (9, 86, 145, 146).

### VUE: Clinical Implications and Recurrence Risk

The majority of pregnancies affected by VUE end in live birth: 73/78 (94%) from a study in 2016, in which the average gestational age at birth was 36.7 weeks (25). However, it has been associated with recurrent pregnancy loss in small series and clinical severity appears to increase with recurrence (26, 27, 33). When VUE is associated with adverse outcome, the principal feature is fetal growth restriction, which occurs in up to two-thirds of cases (25, 28–30). VUE has also been associated with perinatal mortality, necrotising enterocolitis and abnormal neurodevelopmental outcome (143, 147, 148). The risk of recurrent VUE ranges from 17-54% (27, 33, 34) in individual studies but is generally quoted as 10-15% in expert review articles (9, 12).

Estimating the recurrence rate of VUE is challenging. The primary issue is that its incidence is very difficult to precisely determine: general (as opposed to specialist perinatal) pathologists commonly under-diagnose VUE (149) and its focal distribution means that insufficiently thorough placental sampling will lead to missed cases (12). Altemani et al. demonstrated that six blocks are required to detect 85-95% of cases of VUE, while using four blocks could lead to over 30% of cases being missed (150). Even if adequate sampling is performed, there is significant inter-observer variability even among experienced pathologists (151). These diagnostic difficulties are compounded by the small numbers and retrospective design in the majority of available studies. Attempts to determine recurrence rate can only be truly accurate if every subsequent pregnancy of each affected woman is studied, which is logistically unfeasible.

Two small cohorts have reported recurrence rates of 10/59 (17%) (33) and 7/19 (37%) (27). However, a much larger study designed specifically to investigate recurrence risk in VUE found a rate of 102/190 (54%) among 883 women who had had two placentas submitted for histological analysis during the study period (34). The significantly higher rate identified in this study likely reflects selection bias: only singleton live births in which there was a clinical indication for placental histology (e.g. FGR, severe pre-eclampsia, placental abruption) were included. As the authors point out, low-grade VUE that occurs with normal pregnancy outcome may be less likely to recur; and these placentas would not have been included in the cohort (34).

The extent to which VUE causes FGR is, like its recurrence risk, difficult to ascertain. The high prevalence of VUE in term placentas with normal pregnancy outcome (54) suggests it could represent a physiological process that develops as tolerance wanes. However, VUE is consistently over-represented in cohorts of growth-restricted infants, implying a potential contribution to pathology (28, 152–154). Becroft et al. detected VUE in 88/509 (17%) placentas from infants with birthweight <10^th^ centile vs. 62/529 (12%) placentas from appropriately-grown infants (145). This raises an important question: how could this common histological observation cause adverse outcome in a small minority, and account for high proportions of FGR? Some of this effect can be attributed to the grade of VUE: studies using methodology that only detects severe VUE are likely to report higher incidences of adverse outcomes. This is consistent with evidence that higher-grade VUE is more strongly associated with FGR and stillbirth than low-grade VUE (27).

Severe CHI and MPFD can lead to complete obliteration of the intervillous space through accumulation of CD68+ histiocytes and/or fibrin, respectively. It is easy to perceive how this leads to fetal hypoxia, growth restriction and demise (20, 55). However, the same cannot be said for VUE. Even in the most severe high-grade lesions, it is very rare for more than 10% of the chorionic villi to be affected (9). It seems surprising that involvement of such a small fraction of the placental parenchyma could compromise fetal growth (145). It may therefore be the fetal inflammatory response, rather than the percentage of the placental parenchyma infiltrated by maternal immune cells, that determines clinical outcome.

This hypothesis is supported by the discovery of elevated CXCL10 levels in fetal plasma in 22 pregnancies affected by VUE compared to 22 normal term pregnancies (108). Increased CXCL10 is a hallmark of rejection in renal and cardiac allografts (155–157). These findings reinforce the theory that VUE represents maternal rejection of the placenta, but larger studies are required.

Although more common at term, VUE has also been detected in preterm placentas (27, 29, 34). This could represent either accelerated decline of maternal tolerance, or a specific “rejection” event, but has not been specifically studied. Detailed comparisons of term and preterm VUE using advanced techniques such as placental transcriptomics may help to overcome the gaps in our knowledge.

Once we can distinguish physiological VUE at term from pathological preterm VUE with fetal growth restriction, we should be able to determine risk of recurrence more accurately. Until then, the broad estimates quoted above will remain frustrating for clinicians and patients alike. They highlight how little we know about VUE and the ways in which this common lesion can occasionally account for severe obstetric complications.

### VUE: Evidence for an Immune Aetiology

**Figure 3:**
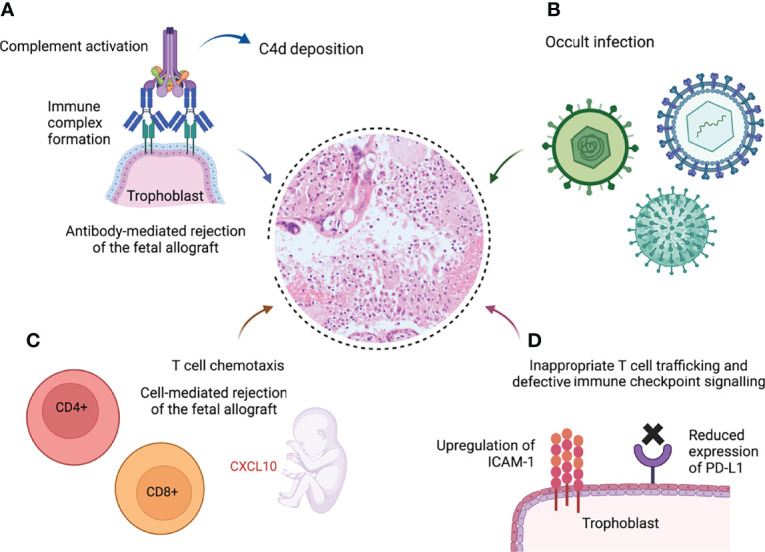
Schematic demonstrating potential pathological pathways leading to villitis of unknown etiology (VUE) (9, 86, 107, 108, 159, 160). **(A)** Formation of immune complexes at the syncytiotrophoblast leads to complement activation and C4d deposition. **(B)** Undiagnosed infection is responsible for the chronic inflammation. **(C)** The fetal response to maternal infiltration of the villi includes elevated plasma levels of the T cell chemokine CXCL10, a hallmark of solid organ allograft rejection (8). **(D)** Excessive intercellular adhesion molecule-1 (ICAM-1) expression on the syncytiotrophoblast in VUE promotes infiltration of maternal T cells (159); programmed death ligand-1 (PD-L1), which suppresses cytotoxic T cell activity, is downregulated in VUE (160). Created with BioRender.com.

Current hypotheses around the pathogenesis of chronic inflammation in VUE are divided into 2 categories: firstly, a response to an as yet unidentified infection; and secondly, a primary immunological mechanism analogous to allograft rejection (158). The latter theory will be the focus of this section. Evidence is summarised in **Figure 3**.

Linear C4d deposition has been detected on the syncytiotrophoblast of VUE placentas, but was absent in healthy placentas and those infected with cytomegalovirus (71, 161). The fact that VUE is significantly more common in donor-oocyte pregnancies (where the fetus is completely allogeneic) compared with own-oocyte IVF pregnancies lends support to the humoral rejection hypothesis (162). However, the placental alloantigen to which the mother raises an antibody, triggering complement activation, is unknown.

The HLA system on the semi-allogeneic placenta is a possible target, just as circulating anti-HLA antibodies are a major risk factor for graft rejection in transplant recipients (163). Villous trophoblast typically lacks HLA class II expression, but VUE placentas demonstrate abnormal upregulation of HLA class II molecules (158, 164). A group in Michigan found a significantly higher frequency of maternal anti-fetal-HLA seropositivity in pregnancies affected by VUE compared to those without: anti-HLA class I antibodies were present in 39/53 (74%) vs. 74/227 (33%), and anti-HLA class II antibodies were present in 23/53 (43%) vs. 29/227 (13%). The same findings were observed for chronic chorioamnionitis, which, like VUE, involves maternal cellular infiltration of fetal tissues – in this case, the chorio-amniotic membranes, rather than the placental parenchyma (165).

As well as humoral mechanisms, the possibility of cellular-mediated rejection has been explored. Kim et al. demonstrated significant upregulation of chemokines and chemokine receptors in 20 VUE placentas, compared to controls (108). The transcriptomic signature they identified is similar to that found in rejected allografts (166–168). These findings were corroborated in a larger study by the same group (169) and also in a report by Enninga et al., who found that 38% of the differentially expressed genes in VUE placentas as compared to normal gestational-age-matched control placentas mapped to allograft rejection pathways and that CXCL10 was the most strongly upregulated gene (158). The observation of increased T-regs in VUE infiltrates vs. normal placentas could indicate a compensatory attempt to suppress inflammation and restore the tolerogenic state (170).

Inappropriate T cell trafficking is another potential contributing mechanism in the pathogenesis of VUE. In healthy pregnancy, physiological suppression of intercellular adhesion molecule-1 (ICAM-1) expression helps to maintain tolerance towards the fetus (171). In VUE, however, ICAM-1 is upregulated on the syncytiotrophoblast, promoting transfer of maternal lymphocytes into the villi (159).

Defects in tolerogenic immune checkpoint signalling pathways may also have a role. Programmed death ligand-1 (PD-L1), a major suppressor of cytotoxic T cells that is pivotal in tumour immune evasion (172) and may be a key player in fetal tolerance (173), is expressed on the trophoblast in normal pregnancy (174) and circulates in maternal blood (175). Shahi et al. found that PD-L1 expression was significantly reduced in VUE placentas compared to both normal and CMV-infected placentas, suggesting a loss of tolerance unique to non-infectious villitis that may play a critical role in the disease process of VUE (160).

## Chronic Histiocytic Intervillositis (CHI)

### CHI: Definition, Prevalence, and Diagnosis

CHI is defined as a CD68+ maternal histiocytic infiltrate occupying ≥5% of the intervillous space (22). This is accompanied by a small population of CD4+ and CD8+ T cells, varying degrees of perivillous fibrin deposition, villous agglutination and trophoblast necrosis (94, 176–178).

CHI is much less common than VUE. A large Canadian series analysed almost 30,000 placentas and reported a prevalence of 0.17% (10). However, CHI and VUE frequently coexist (179): in the first account of CHI published by Labarrere & Mullen in 1987, it was described as an “extreme variant” of VUE (17). Nowak et al., who reported outcomes from 76 cases of combined CHI-VUE, suggested that CHI might represent either a precursor lesion or a more aggressive variant of VUE (25). Although Labarrere later described phenotypic similarities in the cellular infiltrates (180), current consensus is that they represent two distinct syndromes, with the chronic inflammation of CHI confined to the intervillous space rather than infiltrating into the villi (13).

In 2018, Bos et al. proposed diagnostic criteria for CHI as follows: ≥5% of the intervillous space occupied by a mononuclear cell infiltrate, of which ≥80% are CD68+, in the absence of clinical or histopathological signs of infection (22). These have been widely adopted since and recently adapted to produce a grading system for lesion severity that correlated with overall perinatal survival in a cohort of 122 cases (181). Concurrent MPFD has been omitted from these criteria as grading of MPFD intensity is highly subjective and evidence regarding its correlation with clinical outcomes is conflicting (23, 55, 181, 182). Its reported incidence alongside CHI varies widely, from 16% (55) to 100% (176). In a recent large series, 51/111 (46%) of CHI cases were associated with MPFD (56).

### CHI: Clinical Implications and Recurrence Risk

Unlike VUE, CHI is consistently associated with all forms of adverse pregnancy outcome (22, 25, 56). In a systematic review of 350 CHI pregnancies in 291 women, the live birth rate was just 135/246 (55%) overall. Fetal growth restriction affected 63/88 (72%), first-trimester miscarriage 62/256 (24%) and stillbirth 59/155 (38%) (22). Another series of 122 cases reported that 18/122 (15%) ended in medical termination due to severe FGR. In this cohort, 38/70 (54%) of live births were delivered preterm (from 24 to 36 + 6 weeks’ gestation) (35).

The risk of recurrent CHI is high. However, reports of the exact recurrence rate vary from 25-100% in large, well-phenotyped cohorts (10, 22, 35–37, 94, 181, 183, 184). These disparities result from multiple factors (1): the rarity of the lesion and its variable appearance in different trimesters (2); the fact that some studies have excluded first-trimester miscarriages (3); under-diagnosis of CHI due to lack of familiarity even among specialist perinatal pathologists (4); the lack (until recently) of consensus diagnostic criteria (5); the influence of selection bias in small case series of adverse outcomes; and (6) the recent observation that CHI secondary to infection, in particular SARS-CoV-2, does not seem to recur (12, 22, 56, 82, 185).

### CHI: Evidence for an Immune Aetiology

**Figure 4:**
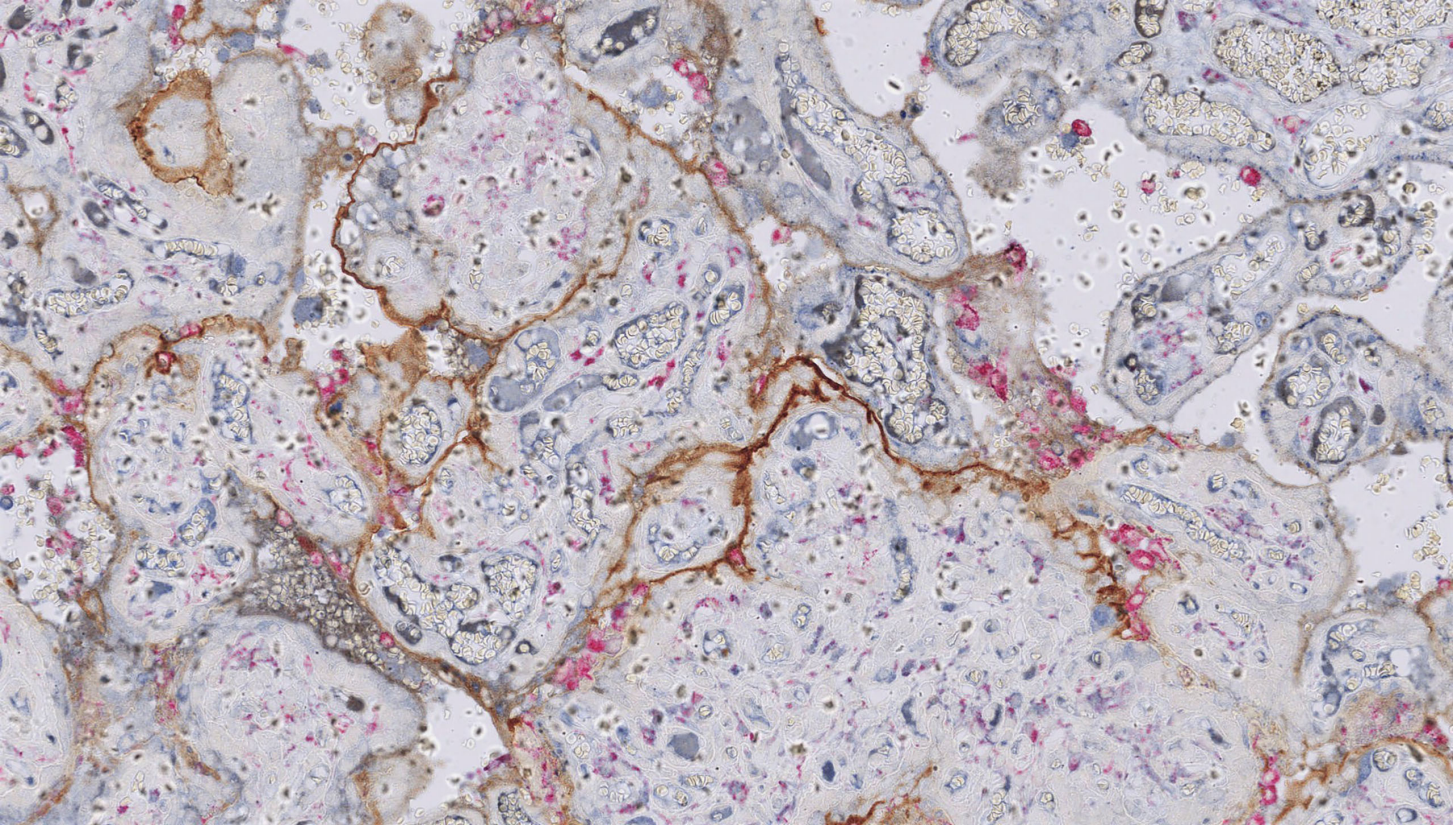
Chronic histiocytic intervillositis (CHI). Dual staining demonstrates aggregates of CD68+ histiocytes in the intervillous space (pink) and linear deposition of C4d along the microvillous border (brown) in a case requiring delivery by emergency Caesarean section for severe fetal growth restriction with abnormal umbilical artery Dopplers at 24 weeks’ gestation. Histology also showed extensive perivillous fibrin deposition.

Investigations into a potential immune aetiology for CHI largely mirror those conducted in VUE. C4d deposition on the syncytiotrophoblast is emerging as a consistent feature of CHI. The percentage of villi that stain positive for C4d is significantly higher in CHI placentas than in normal controls (182) and in VUE (72). The authors hypothesise that complement fixation localised to the microvillous border could represent a maternal humoral response to a paternally inherited placental alloantigen. This could induce expression of pro-inflammatory cytokines that recruit maternal macrophages into the intervillous space (72). However, placental C4d deposition is also evident in other autoimmune conditions such as SLE and APS, which can coexist with CHI (116). While the presence of C4d reinforces the theory that CHI results from humoral immune rejection, it does not of course identify the causative placental antigen. Benachi et al. confirmed diffuse villous staining for C4d in recurrent CHI and also noted focal deposition of the C5b-9 membrane attack complex, suggesting localised complement activation (83). **Figure 4** shows C4d deposition in CHI.

In parallel with VUE, one study showed increased frequency of partner-specific anti-HLA antibodies in women with CHI (3/4, 75%) compared to controls (0/7) (84). However, the numbers studied were very small (only 4 affected women). Moreover, formation of anti-partner-HLA antibodies is also seen in normal pregnancy and is a physiological maternal response to peri-conceptional exposure to paternal antigens (13, 186) and term birth (187).

An important and detailed immunophenotypic analysis of two women who had healthy full-term first pregnancies each followed by two subsequent pregnancies with histologically and clinically worsening CHI supports the theory that CHI is driven by an alloimmune mechanism. In both cases, anti-HLA antibodies that showed specific reactivity for epitopes harboured by all 3 fetuses were isolated. The authors concluded that the first pregnancy constitutes a sensitising event that establishes broad humoral reactivity against HLA-eplet-mismatched siblings (83). Although small, this study provides evidence of alloimmune sensitisation in CHI. A larger-scale study would be required to validate these findings.

Freitag et al. used placental transcriptomics to analyse inflammation- and angiogenesis-associated gene expression in 5 CHI placentas, 4 VUE placentas and 7 normal control placentas. Compared to controls, the CHI group showed significantly higher levels of transforming growth factor beta receptor 1 (TGFBR1) and matrix metallopeptidase-9 (MMP-9) (178). Although there is evidence that macrophage trafficking depends on MMP-9 (188), it is impossible to conclude from such a small cohort that MMP-9 overexpression drives histiocyte accumulation in CHI.

ICAM-1 expression is markedly higher in CHI than both VUE and control placentas (189). However, ICAM-1 promotes adhesion and transmigration of maternal cells into fetal tissues – for its expression to be higher in CHI, where maternal cells remain confined to the intervillous space, than in VUE, where maternal cells infiltrate and destroy the villi, therefore seems counter-intuitive. It is likely that increased ICAM-1 expression is sufficient to arrest maternal cells in the intervillous space, but whether or not they then invade across the syncytiotrophoblast barrier into fetal tissues depends on the cellular composition and properties of the infiltrate itself.

Differential expression of two other cell surface proteins in CHI has recently been reported. Firstly, there was a significant reduction in the percentage of villi staining positive for CD39 in 22 CHI placentas compared to 20 healthy controls (45% vs. 95%) (182). CD39 is a key contributor to T-reg-mediated tolerance and is expressed at lower levels in a wide range of autoimmune diseases (190). In this study, the extent to which CD39 was downregulated correlated directly with clinical outcome, with greater reductions in the subgroup of those with CHI who had FGR or intrauterine death. However, the usual caveat of poor quantification of protein expression when ascertained through immunostaining alone should be applied here (182).

Secondly, Clark et al. showed a significant reduction in CD200 expression intensity on villous trophoblast from a single CHI placenta compared to a healthy term control (191). CD200 is an anti-inflammatory immune checkpoint molecule that inhibits macrophage proliferation and promotes T-reg polarisation through interaction with its receptor, CD200R. CD200-/- knockout mice develop expansion of activated macrophage populations and increased susceptibility to autoimmunity (192, 193). However, there are a number of caveats to the study demonstrating reduced CD200 in CHI: firstly, its size (n=1); secondly, CD200R expression was also reduced; and thirdly, these reductions were only seen in villous trophoblast but not in the intervillous infiltrate (191). Although a mechanism of CD200 depletion appears consistent with the pathophysiology of CHI, this result should therefore be interpreted with caution.

The aggressive clinical phenotype of CHI is at odds with the characteristics of the histiocytic infiltrate. The maternal CD68+ macrophages that occupy the intervillous space overexpress CD163, indicating polarisation towards the anti-inflammatory M2 phenotype associated with matrix remodelling and tissue repair (194). This is consistent with the lack of fetal tissue infiltration, but inconsistent with the severity of the adverse obstetric outcomes in CHI. It is possible that the intervillous infiltrate is a consequence of an as yet undiscovered immune pathology, rather than the primary driver of disease.

## Massive Perivillous Fibrin Deposition (MPFD)

### MPFD: Definition, Prevalence, and Diagnosis

The term MPFD describes a pathological process in which >25% of the intervillous space is occupied by fibrin. The fibrin forms a lattice that suffocates the chorionic villi and impairs feto-maternal gas exchange. This is accompanied by foci of degenerative trophoblastic injury (20, 21). It can be categorised as classic, transmural or borderline according to the site and extent of deposition (see **Table 1**) (19).

In isolation, MPFD is uncommon. The largest published cohort found MPFD in 190/39,215 (0.48%) of placentas sent for histopathology (11) and the incidence is approximately 0.28 cases per 1,000 live births (21). As with VUE and CHI, estimations of prevalence are fraught with inaccuracy because of selection bias: histopathological examination of the placenta is not routine and is only conducted when adverse perinatal outcomes occur. Despite this uncertainty, it is clear that the frequency of MPFD varies with gestational age. Recent data demonstrate higher rates in first-trimester miscarriages: 2.7% (15/562) (73).

MPFD often overlaps with VUE/CHI rather than occurring as an isolated lesion, and how this influences clinical outcomes is difficult to ascertain. One study examined 318 first-trimester miscarriage specimens with normal karyotype and identified isolated CHI in 14/318 (4.4%), isolated MPFD in 17/318 (5.3%) and a combination of chronic inflammation with MPFD in 60/318 (19%) (195).

### MPFD: Clinical Implications and Recurrence Risk

The reported risk of fetal death in MPFD ranges from 15-80% (11, 21, 31, 32, 109, 196). In general, studies quoting higher rates are smaller: 24/60 (40%) and 4/10 (80%) (31, 109). Studies analysing >50 cases quote lower rates that are likely to be more reliable: 32/190 (17%) (11) and 11/71 (15%) (32). This broad range is unhelpful for clinicians and makes it difficult to counsel patients appropriately. As with VUE and CHI, several factors contribute to this challenging ambiguity: small studies, subjectivity of reporting, a lack of consensus on diagnostic criteria and terminology, and failure to distinguish infectious vs. idiopathic cases.

MPFD is also associated with high rates of prematurity (26-58%) (11, 21, 31, 32) and fetal growth restriction (53-88%) (31, 32, 38, 197). It is independently associated with overall severe adverse neonatal outcome (198) and, in preterm infants, significantly higher rates of neurodevelopmental impairment at 24 months than in gestational age-matched cases without MPFD (197). Pregnancy outcomes appear to be worse with higher degrees of fibrin deposition. Intrauterine death, preterm birth and birthweight <3^rd^ centile for gestational age were all significantly more common in a study comparing 39 cases of severe transmural MPFD to 32 cases of borderline MPFD and 142 controls (32).

The reported risk of recurrence in MPFD is highly variable and ranges from 12-78% (11, 19, 21, 31, 38, 39). As with VUE and CHI these statistics are of uncertain accuracy given the subjectivity of the diagnosis, the failure to consistently send placentas from subsequent pregnancies for histology and the exclusion of miscarriages from some studies (19, 21, 32).

### MPFD: Aetiology

Theories about the aetiology of MPFD extend more broadly than those for VUE and CHI. As stated above, small amounts of fibrin deposition are a physiological finding in term placentas and reflect activation of the maternal coagulation cascade in response to turbulent intervillous blood flow (20, 199). However, the shift from physiological to pathological fibrin deposition that defines MPFD can arise in response to a large number of different insults. These include infection, maternal autoantibodies that provoke local complement activation, maternal thrombophilia, or direct cytotoxicity (which arises from excessive proliferation of extravillous trophoblast, leading to overproduction of major basic protein) (20, 200). All these sources of trophoblast injury could lead to localised inflammation, stasis within the intervillous space, excessive activation of the coagulation pathway, and development of MPFD either in isolation or accompanied by VUE and/or CHI (80, 199, 201).

Accumulation of damaged and degenerating trophoblast exposes the basement membrane to the intervillous blood. This activates the coagulation system and leads to synthesis of fibrinoid extracellular matrix, which occludes the intervillous space (20, 21). As well as a specific response to trophoblast injury, MPFD could also represent the terminal phase of a secondary response to progressive trophoblast damage resulting from severe destructive villitis or intervillositis, since they often occur together (195). These theories are appealing given the frequent coexistence of the 3 disorders in question. However, the “snapshot” nature of placental pathology makes the temporal relationship between the lesions difficult to establish.

Determining the source of trophoblast injury is fundamental to the understanding of MPFD. In maternal infection, haematogenous spread brings pathogens into the intervillous space where they can cause direct trophoblast damage. MPFD has been reported in conjunction with enteroviruses, cytomegalovirus, SARS-CoV-2 and syphilis (100, 202–205). In one of these reports, a patient who had an intrauterine death at 36 weeks’ gestation with MPFD (>80% of the intervillous space occupied) and Coxsackievirus A16 infection of the placenta went on to have an uneventful pregnancy 15 months later, though the placenta was not sent for histology (205). Perhaps unsurprisingly, infection-associated MPFD does not appear to recur (202, 205). It is the cause and management of recurrent cases in the absence of infection that pose the greatest challenge for clinicians.

Gogia et al. found a high prevalence of maternal thrombophilia in cases of MPFD, particularly protein S deficiency, which was present in a total of 9/26 (35%) of women tested 3 months after the pregnancy had ended. They speculated that this might promote dysfunctional activation of the coagulation system in the intervillous space (206). However, this was not replicated in a larger study in which none of the 71 women affected by MPFD had a thrombophilia (though the type being tested for was not specified) (32).

In parallel with their work on VUE, the Michigan group have demonstrated significantly increased rates of C4d deposition, maternal anti-fetal-HLA class I seropositivity and upregulation of the T cell chemokine CXCL10 in maternal plasma in women affected by MPFD, albeit in small numbers (73, 109, 207). In their 2013 study, they compared levels of plasma CXCL10 in serial antenatal samples taken every 2-4 weeks from women subsequently found to have MPFD (n=10) and controls who delivered a healthy term infant with normal placental histology (n=175). They demonstrated a longitudinal rise in plasma CXCL10 with advancing gestational age in women with MPFD. In contrast, CXCL10 levels declined steadily from 16 weeks’ gestation in controls and were significantly lower than in women with MPFD by 20 weeks’ gestation. This led the authors to speculate that CXCL10 could constitute an antenatal predictor for the development of MPFD (109). Although this finding holds some potential given the current lack of a reliable antenatal biomarker for MPFD, systemic elevation of a T cell chemokine cannot explain the fact that the pathology is limited to the intervillous compartment.

The prevalence of autoimmune disease in MPFD has not been well quantified. MPFD has been reported in conjunction with SLE and APS (32, 73, 199). This is consistent with an antiphospholipid antibody-induced decrease in trophoblast expression of the anticoagulant annexin-V, promoting a hyper-coagulable state within the placenta that could lead to excessive fibrin deposition (64). However, the most consistent association of MPFD is with polymyositis (201, 208–210). Autoantibodies, particularly anti-Jo-1, are detectable in the majority of cases of polymyositis and it is associated with adverse pregnancy outcome (201, 209). Skeletal muscle and placental syncytiotrophoblast both possess the unusual ability to form syncytia. It is possible that a shared antigen triggers T cell-mediated attack on both tissues simultaneously, accounting for the apparent association between polymyositis and MPFD (210).

## Immunosuppression To Prevent Recurrent Adverse Pregnancy Outcome

There is very little evidence available around optimal treatment approaches for prevention of recurrent adverse pregnancy outcomes due to chronic inflammatory placental disorders. The suspected alloimmune contribution to the pathogenesis of CHI and VUE has prompted case reports and small series describing variable clinical benefit in response to prednisolone, hydroxychloroquine, tacrolimus, intravenous immunoglobulin and adalimumab (10, 37, 73, 82, 184, 211–214). However, drug combinations and dosing regimens are highly variable and results are conflicting.

Early small studies of heterogeneous combinations of thromboprophylaxis and immunosuppression showed no reduction in adverse pregnancy outcomes (37). However, more organised and rational immunosuppressive regimens have shown promise.

A prospective study in 2015 reported on 24 patients with previous CHI who underwent subsequent pregnancies treated with various combinations of aspirin, LMWH, prednisolone and hydroxychloroquine. The live birth rate for the treated pregnancies was 16/24 (67%) overall, a significant rise from 24/76 (32%) in their previous pregnancies. The authors drew attention to the fact that 4/6 women receiving hydroxychloroquine had a live birth (184). However, larger and more recent studies are less confident in endorsing treatment: a French cohort of 122 cases found no evidence for benefit from aspirin (n=18), LMWH (n=7) or prednisolone (n=6) in comparison to the 90 untreated cases – but again, doses were not specified (56). Despite the lack of conclusive evidence, some authors now advocate routine consideration of immunosuppression (10) while others propose enhanced obstetric surveillance alone (56). These small-scale contradictory reports have created considerable dilemmas for both patients and providers.

The largest cohort of women with previous CHI treated in 1-2 subsequent pregnancies is 39, with 27/39 (69%) receiving prednisolone and/or hydroxychloroquine. This combination significantly reduced histological severity of CHI: in the index pregnancy, 11/28 (39%) cases were mild, 11/28 (39%) were moderate and 6/28 (21%) were severe; whereas in subsequent pregnancies treated with prednisolone and/or hydroxychloroquine, CHI was absent in 18/30 (60%) and only severe in 2/30 (7%). It also translated into a non-significant trend towards higher rates of live birth in the prednisolone and/or hydroxychloroquine group, compared with those who received non-immunomodulatory treatment: 25/29 (86%) vs. 8/13 (62%), p=0.11 (179).

## Challenges And Limitations

Clinical studies that have examined the treatment of chronic inflammatory placental disorders have obvious limitations. They use small numbers, lack randomisation and include heterogeneous patient groups with poorly defined histopathology. This reflects the lack of research into the conditions and the tendency to rely on low-quality studies. Additional caveats in the primary literature about VUE, CHI and MPFD include:

1) Proven significant inter-observer variability in the diagnosis of the three placental disorders in question (12, 23, 151, 181, 215);
2) Discrepancies in the definitions used for adverse clinical outcomes: there is no consensus in the data on what constitutes “early” or “late” miscarriage, whether FGR is defined as estimated fetal weight below the 3^rd^ or 10^th^ percentile for gestational age, and the method used to calculate these centiles;
3) The absence of clear, validated consensus criteria for stratification of lesion severity and the strength of its correlation with clinical outcome (10, 22, 23, 181);
4) The confounding effect of aspirin and low-molecular weight heparin, which are frequently recommended in women with recurrent adverse pregnancy outcome irrespective of the cause, in determining the role of maternal immunosuppression (5, 216);
5) The extent to which general improvements in obstetric care have influenced clinical outcomes: the UK stillbirth rate has fallen from 5.5 per 1,000 total births in 1985, when many of the early series describing VUE/CHI/MPFD were published, to 3.8 per 1,000 total births in 2019 (217). The global stillbirth rate is estimated to have fallen by 35% since 2000 (218). Alongside this, increased obstetric surveillance and various forms of treatment for women with VUE, CHI and MPFD have become the norm in several countries and it is difficult to ascertain which of these, if any, lead to improved outcome (179).

The availability and utilisation of perinatal pathology is another major challenge. VUE, CHI and MPFD are difficult to diagnose. Despite some limited evidence for an association with antenatal biochemical [raised serum alkaline phosphatase (35, 56), low PAPP-A (181)] and ultrasonographic [abnormal umbilical artery Doppler, oligohydramnios (56, 185)] biomarkers, these are non-specific and placental histopathology remains the only reliable method for diagnosis. But even in women with recurrent disease, specimens are still not reliably sent for histopathology. In the cohort of 56 CHI cases described by Simula et al. in 2020, there were 36 subsequent pregnancies but less than half (14/36) had placental analysis to assess for recurrent CHI (10). Availability of specialist perinatal pathology services is subject to financial constraints and regional disparities, exacerbated by the recent COVID-19 pandemic. Many obstetric units use their own local eligibility criteria, with variable adherence (179, 219, 220). This makes accurate quantification of recurrence risk and patient counselling extremely challenging.

Affected patients are well-informed, highly motivated, increasingly seek immunosuppressive therapy and are unlikely to find the prospect of an untreated pregnancy acceptable. Ensuring appropriate referral for histopathological analysis of the placenta is therefore paramount if we are to narrow the spectrum of reported recurrence rates and understand treatment efficacy in these conditions. We consider placental pathology essential for all women with a history of CHI, VUE, MPFD and/or SARS-CoV-2 infection in pregnancy, as it is for all cases of fetal growth restriction or demise.

## Current Research Gaps

Several unanswered questions remain in our knowledge of these conditions:

1. The histological features of VUE, CHI and MPFD can all be seen in normal pregnancy. Do the three conditions simply represent exaggerated forms of normal responses to advancing gestational age or to placental injury? Where along the continuum do we designate pathology, and what triggers the transition from physiology to disease?
2. Are there different clinical phenotypes of VUE/CHI/MPFD in infection-associated compared with immune-associated cases?
3. Do VUE, CHI and MPFD represent manifestations of maternal “rejection” of the feto-placental unit? If yes, are they antibody- or T-cell-mediated, what determines which disorder develops, and how does the fetal inflammatory response contribute to disease?
4. Exclusion of an infectious aetiology is essential to determine whether or not VUE, CHI and MPFD are likely to recur. How reliable is our screening for placental infection? How might new techniques help to exclude occult infection?
5. What is the mechanism underlying recurrent disease? Do women become sensitised against a specific placental antigen in a first pregnancy, or does recurrence arise due to non-specific cross-reactivity with antigens encountered during infection or exposure to a chromosomally abnormal pregnancy?
6. Is massive perivillous fibrin deposition a “final common pathway” in a non-specific response to trophoblast damage that obscures the initial pathology as it accumulates?
7. What is the role of maternal immunosuppression in preventing recurrence of VUE, CHI and MPFD? Which are the most effective drug regimens, and how should these be integrated with obstetric surveillance in a subsequent pregnancy?

## Conclusion

The three placental disorders discussed in this article are strongly associated with recurrent adverse perinatal outcomes but remain incompletely understood and difficult to treat. Accumulating evidence in favour of an alloimmune “fetal rejection” aetiology and an increasing recognition of their clinical implications are likely to prompt wider experimentation with immunosuppressive therapy, but caution is essential until the pathogenesis of these syndromes is firmly established. Physiological gestational immune modulation already predisposes pregnant women to disproportionately severe clinical consequences of infection with SARS-CoV-2, among others (96). Systemic maternal immunosuppression in pregnancy should therefore only be initiated after careful consideration.

Given their rarity and severity, there is a clear imperative for international collaboration and application of novel scientific techniques in the investigation of exactly how CHI, VUE and MPFD cause adverse pregnancy outcome and how this might be prevented. A prospective registry of women diagnosed with these conditions would provide a highly valuable resource to determine whether and how their subsequent pregnancies are affected. We are in the process of establishing a multicentre network of clinicians and scientists interested in chronic inflammatory placental disorders in order to achieve this goal.

The recent discovery of their association with SARS-CoV-2 has given these neglected disorders new prominence in the obstetric community and in the medical literature. There is an opportunity to capitalise on this momentum, address the gaps in our knowledge and collaborate on larger, prospective studies that will elucidate the underlying mechanisms of disease.

## Author Contributions

EC and DW conceived the article. EC wrote the first draft of the manuscript. TM and DW provided critical revisions and edited the text. All authors contributed to manuscript revision, read, and approved the submitted version.

## Funding

EC is supported by an MRC Clinical Research Training Fellowship (MR/V028731/1) that funds investigation into the pathogenesis of chronic histiocytic intervillositis. TM is supported by an MRF fellowship (MRF-057-0004-RG-MCDO-C0800). DW is supported by the National Institute for Health Research University College London Hospitals Biomedical Research Centre.

## Conflict of Interest

The authors declare that the research was conducted in the absence of any commercial or financial relationships that could be construed as a potential conflict of interest.

## Publisher’s Note

All claims expressed in this article are solely those of the authors and do not necessarily represent those of their affiliated organizations, or those of the publisher, the editors and the reviewers. Any product that may be evaluated in this article, or claim that may be made by its manufacturer, is not guaranteed or endorsed by the publisher.
